# Mosquito Behavior Change After Distribution of Bednets Results in Decreased Protection Against Malaria Exposure

**DOI:** 10.1093/infdis/jiw615

**Published:** 2016-12-22

**Authors:** Edward K. Thomsen, Gussy Koimbu, Justin Pulford, Sharon Jamea-Maiasa, Yangta Ura, John B. Keven, Peter M. Siba, Ivo Mueller, Manuel W. Hetzel, Lisa J. Reimer

**Affiliations:** 1 Liverpool School of Tropical Medicine, United Kingdom; 2 Papua New Guinea Institute of Medical Research, Madang; 3 Papua New Guinea Institute of Medical Research, Goroka; 4 Michigan State University, East Lansing; 5 Walter and Eliza Hall Institute, Parkville, Victoria, Australia; 6 Swiss Tropical and Public Health Institute, Basel, Switzerland; 7 University of Basel, Switzerland

**Keywords:** Infectious Disease Vectors, Mosquito Control, Mosquito behavior, Insecticide-Treated Bednets, Malaria

## Abstract

**Background.:**

Behavioral resilience in mosquitoes poses a significant challenge to mosquito control. Although behavior changes in anopheline vectors have been reported over the last decade, there are no empirical data to suggest they compromise the efficacy of vector control in reducing malaria transmission.

**Methods.:**

In this study, we quantified human exposure to both bites and infective bites of a major malaria vector in Papua New Guinea over the course of 4 years surrounding nationwide bednet distribution. We also quantified malaria infection prevalence in the human population during the same time period.

**Results.:**

We observed a shift in mosquito biting to earlier hours of the evening, before individuals are indoors and protected by bednets, followed by a return to preintervention biting rates. As a result, net users and non–net users experienced higher levels of transmission than before the intervention. The personal protection provided by a bednet decreased over the study period and was lowest in the adult population, who may be an important reservoir for transmission. Malaria prevalence decreased in only 1 of 3 study villages after the distribution.

**Discussion.:**

This study highlights the necessity of validating and deploying vector control measures targeting outdoor exposure to control and eliminate malaria.

In the past 2 decades, global efforts to reduce the burden of malaria have intensified. Since 2000, the primary strategy to limit transmission has been the distribution of insecticide-treated bednets (ITNs). Recent estimates suggest that in the last 15 years, ITNs have been responsible for preventing 68% of the 663 million cases that have been averted in sub–Saharan Africa due to increased malaria control efforts [1]. However, it is well recognized that strategies solely targeting endophagic, anthropophagic, and endophilic vectors may not be sufficient to control and eliminate malaria [2]. This is particularly true outside Africa where vectors exhibit greater behavioral plasticity. Control efforts can result in shifts in vector behavior and/or species composition such that the post-intervention vector community is less likely to come in contact with insecticide [3–9].

Studies to accurately quantify exposure to bites and the true protective efficacy of long-lasting insecticidal nets (LLINs) [10] have revealed that the vast majority of exposure still occurs inside, when people can be protected by an LLIN [11]. Thus, despite shifts to outdoor feeding [4, 5, 12] and changes in biting times [13], the personal protection provided by LLINs remains high (>80%). In areas outside of sub–Saharan Africa where vectors bite earlier and outside, LLINs can still reduce transmission through the combined effect of frequent blood-feeding and a homogenous host-seeking phenotype [14, 15]. Regardless, in some settings, evidence suggests that these behavioral changes are decreasing the personal protection against bites offered by an LLIN [16], a worrying prospect for malaria control and elimination in these areas.

It is well established that residual malaria transmission (transmission that remains despite universal coverage of effective interventions [2]), can be intense. However, it is currently unknown whether the interventions that are deployed against malaria vectors have the ability to increase residual transmission intensity through shifts in behavior or how shifts in behavior may impact human infection prevalence. Modeling suggests that the presence of behavioral resistance could dramatically increase transmission, perhaps more so than physiological resistance [8], which is currently poised to create a public health disaster if not confronted immediately. Behavioral resistance could have catastrophic consequences for the sustainability of currently available vector control methods, especially in areas outside of sub–Saharan Africa characterized by outdoor transmission [17, 18]. In this study, we estimate the exposure to infective bites experienced by children and adults in Papua New Guinea (PNG) before and after a nationwide LLIN distribution. In addition, we quantified malaria infection prevalence in the mosquito and human populations. We show that following the intervention, there was a shift in the biting behavior of the major malaria vector, *Anopheles farauti* 4. This caused the protective efficacy of LLINs to decrease, and the ability of nets to control malaria in this situation was compromised.

## METHODS

### Mosquito Collection

Longitudinal monitoring of mosquito abundance was performed by outdoor human landing catch in Kokofine (−5.69029, 145.4801) and Mauno (−5.65079, 145.493) villages in Madang Province of PNG. These villages sit 4.6 km apart in the Ramu River valley. Trained collectors sat outside a house with their pant legs rolled up. They collected host-seeking mosquitoes that landed on their legs with an aspirator, and stored all captured mosquitoes in cups according to hour of collection. One collector worked from 6 pm to 12 am, and another worked from 12 am to 6 am. The collectors switched shifts on sequential collection nights. Two houses were sampled each night, and collections were performed for 6 consecutive nights. The first collections in both villages occurred in December 2008, 1 month before LLINs were distributed in January 2009 [19]. Subsequent collections occurred in November of 2009 and September of 2010. In 2011, collections were performed in March, July, and November, but no significant seasonal variation was observed in either village (in both mean biting rates and infection rates), so results from these 3 months were pooled in subsequent analyses. The species of mosquito was confirmed by polymerase chain reaction–restriction fragment length polymorphism of the ITS2 region [20] using either an individual leg or extracted DNA. Lysates from whole mosquitoes were screened for *Plasmodium falciparum*, *Plasmodium vivax* 210, and *P. vivax* 247 circumsporozoite proteins by enzyme-linked immunosorbent assay [21] in pools of 5 mosquitoes each.

To estimate the proportion of bites experienced inside and outside, additional collections were performed in June 2011. During this month, indoor landing catches were performed simultaneously with the outdoor landing catches at 1 chosen household per night for 6 consecutive nights. The degree of endophagy is presented as the proportion of mosquitoes collected by indoor landing catches out of the paired total.

### Human Behavior

Human movement inside and outside was quantified as part of a national household survey and questionnaire [22]. Heads of household were asked what time individuals in the house went inside, what time they went to bed, and how old they were. Kokofine and Mauno were not included as part of this household survey, but data concerning human movement were similar across the lowland regions of the country. Therefore, patterns of movement and bed times in the Momase region were used in this analysis.

### Human Infection Prevalence

Human infection prevalence was measured in February and March of 2008, 2009, and 2011 in Mauno, Kokofine, and Kesowai (−5.79683, 145.62299) villages. The methods used in this household survey have already been described [19]. Briefly, a finger prick blood sample was taken from consenting individuals aged >5 months old from 30–35 randomly selected households in each village. Stained blood slides were double-read by trained microscopists at the PNG Institute of Medical Research.

### Data Analysis

Nightly biting rates were compared between years using a 1-way analysis of variance and Tukey’s test for post-hoc comparisons. Median biting times, 1st and 3rd quartiles, and 95% confidence intervals were calculated based on the entire catch per village and year. Kruskal–Wallis tests with pairwise comparisons were performed to determine whether the distribution of biting times between years was the same. Sporozoite prevalence was calculated as minimum prevalence, where positive pools were assumed to only have 1 positive mosquito. Prevalence for each year was calculated by dividing the total number of positive pools (with either *P. falciparum* or *P. vivax*) by the total number of mosquitoes in all pools analyzed. Prevalence was compared between years with chi-square tests. At 10 pm, 90% of individuals were inside, so this time point was chosen to compare sporozoite prevalence in early biting mosquitoes using a chi-square test. Four indices of exposure and protection were estimated: exposure to bites (either for a net user [B_p_] or a non–net user [B_u_]), the proportion of exposure occurring indoors (π_i_), the true protection against mosquito bites (P*), and the true protection against infective bites (P*^f^). Estimates of exposure to bites for net users and nonusers were calculated as published previously [10] with 2 modifications. First, because paired indoor and outdoor landing catches were not performed during the entire study period, indoor hourly biting rates were estimated by first calculating the hourly proportions biting inside and outside during the paired collections. Hourly proportions were then multiplied by hourly outdoor biting rates to estimate hourly indoor biting rates. Second, estimates of indoor exposure for net users was refined by accounting for the period after individuals moved inside and before they went to bed. The estimate of exposure for a net user (B_p_) was therefore:

$$B_{P}=\overset{24}{\underset{t=1}{\sum }}\left[B_{o,t}\left(1-I_{t}\right)+B_{i,t}\left(I_{t}-S_{t}\right)+B_{i,t}S_{t}\left(1-P\right)\right],$$

where B_o,t_ is the outdoor biting rate at time t, I_t_ is the proportion of individuals inside at time t, B_i,t_ is the indoor biting rate at time t, S_t_ is the proportion of individuals sleeping at time t, and P is the protection provided by nets, which is assumed to be 0.968 [23]. Similar modifications were made to the calculation of B_u_, P*, and π_i_.

Exposure to infective bites was estimated by first calculating the hourly infection rate N_t_. Exposure to infective bites for a net-user was therefore

$$F_{p}=\overset{24}{\underset{t=1}{\sum }}B_{p,t}N_{t},$$

and for a non-user

$$F_{u}=\overset{24}{\underset{t=1}{\sum }}B_{u,t}N_{t}.$$

The personal protection against infective bites (P^*f^) provided by an LLIN was

$$P^{*f}=1-\frac{F_{p}}{F_{u}}.$$

B_u_ was compared across years in each village using Kruskal–Wallis tests with pairwise comparisons. π_i_ and P* were compared among age groups and years using generalized linear mixed models with a binomial distribution, village as a subject, year by age group as the fixed effect, and household nested within date as a random effect. For each dependent variable, a dataset was constructed using the formulas described herein (or derivatives of) to estimate exposure values for each household and date combination. All statistical analyses were performed with SPSS 22.

Prevalence of malaria positivity was compared between years within each village using chi-square tests.

### Ethical Approval and Informed Consent

Informed consent was obtained from all participants or their parent/guardian for those aged <16 years. This study was approved by the institutional review board at the Papua New Guinea Institute of Medical Research (protocol 0933) and the Medical Research Advisory Council of PNG (protocol 10.12).

## Results

### Biting Rates

Over the course of 4 years (2008–2011), 41757 anopheline mosquitoes were captured by 138 outdoor human landing catch collections. More than 99% (n = 41407) were identified as *An. farauti sensu lato*. The remaining mosquitoes were identified as *An. koliensis* (n = 157), *An. punctulatus* (n = 122), *An. longirostris* (n = 69), and *An. karwari* (n = 2). All 4267 of the *An. farauti s.l*. mosquitoes that were confirmed by polymerase chain reaction were *An. farauti* 4. The nightly biting rate significantly decreased 1 year after LLINs were distributed in both villages (from 560 to 212 bites/person/night in Kokofine, *P* = .001; and from 156 to 37 bites/person/night in Mauno, *P* < .001). In Kokofine, nightly biting rates increased significantly in 2010 (to 374 bites/person/night) and remained at that level in 2011 (418 bites/person/night). In Mauno, nightly biting rates remained low but did increase significantly between 2010 (4 bites/person/night) and 2011 (66 bites/person/night, *P* < .001).

### Host-Seeking Behavior

**Figure 1.**
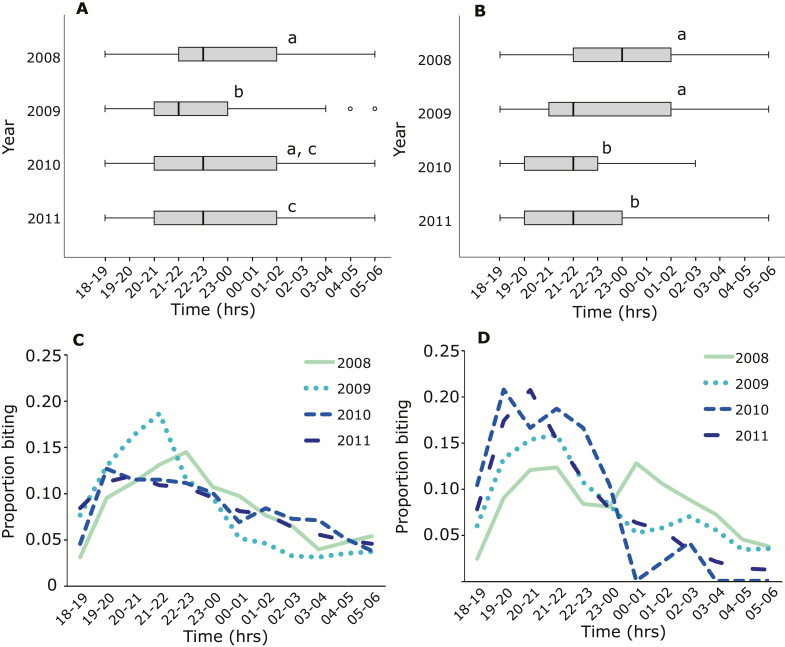
Median outdoor biting times with 1st and 3rd quartiles (boxes) and 95th percentiles (whiskers) in Kokofine (*A*) and Mauno (*B*) villages of Papua New Guinea before (2008) and after (2009–2011) a long-lasting insecticidal net distribution. Years not sharing the same letter indicate significantly different medians using a Kruskal–Wallis test with pairwise comparisons. The proportion of bites occurring at each hour in Kokofine (*C*) and Mauno (*D*) are presented as well.

The median outdoor biting time in both villages occurred significantly earlier after the distribution of LLINs (Figure 1A and 1B). In Kokofine, the median biting time was 11 pm–12am in 2008 and was 1 hour earlier in 2009. Although the value of median biting time returned to 11 pm–12am in 2010–2011, there was still a significant shift from the preintervention value due to the change in bite time distribution, with more mosquitoes biting earlier than the median time after LLIN distribution. In Mauno, the median biting time was 12 pm–1 am in 2008, and shifted 2 hours earlier (10 pm –11 pm) in 2009. In 2010 and 2011, the median biting time remained at 10 pm–11 pm, but the distribution of bites continued to shift even earlier. In both villages, the hour of maximum biting density was 10 pm–11 pm in 2008, and 8 pm–9 pm in 2011 (Figure 1C and 1D). The degree of endophagy remained relatively consistent throughout the hours of the night, with 16.5% of overall bites occurring inside (Supplementary Figure 1).

### Mosquito Infection Prevalence

**Figure 2.**
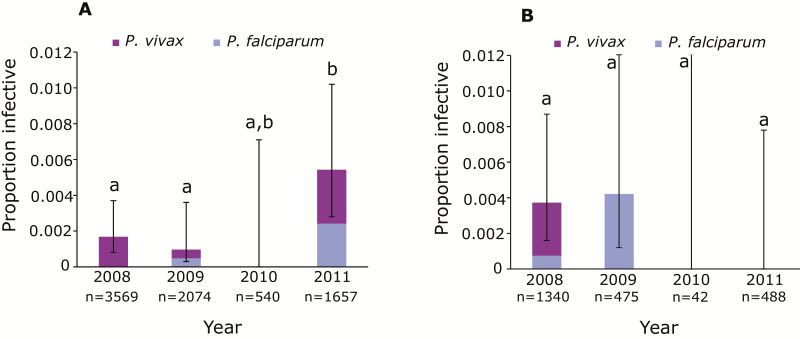
Sporozoite prevalence for *Plasmodium falciparum* and *Plasmodium vivax* in *An. farauti* 4 in Kokofine (*A*) and Mauno (*B*) villages of Papua New Guinea before (2008) and after (2009–2011) a long-lasting insecticidal net distribution. Sample sizes are indicated below each year. Bars not sharing the same letter indicate significant differences using chi-square tests.

Sporozoite prevalence remained consistent across all 4 years in Mauno but increased significantly in 2011 in Kokofine (Figure 2). Mosquito infection prevalence ranged from 0% to 0.54% in Kokofine and 0% to 0.42% in Mauno. The proportion of infective bites occurring before 10 pm was not different between years.

### Human Sleeping Behavior

Movement of people indoors occurred slightly earlier in the highland than in the lowland regions of PNG. Sleeping patterns were similar across the 4 main geographical regions (Supplementary Figure 2).

**Figure 3.**
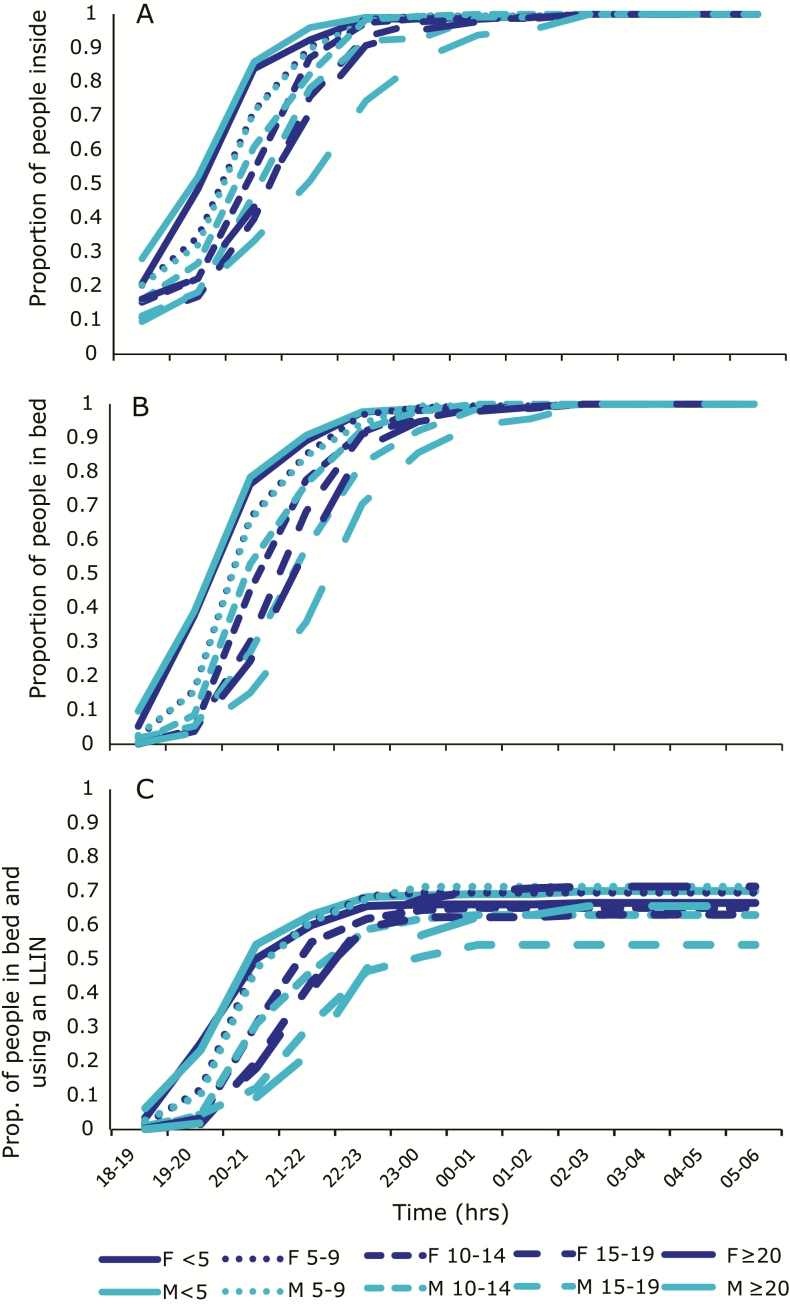
Proportion of males (M) and females (F) from each age group inside (*A*), in bed (*B*), and in bed under a long-lasting insecticidal net (LLIN) (*C*) from 6 pm to 6 am.

In Momase, where Kokofine and Mauno are located, data were disaggregated by sex and age. Adolescent and adult males had later patterns of activity than females, and younger individuals went inside and went to bed earlier than older individuals (Figure 3A and 3B). The proportion of individuals sleeping under an LLIN did not exceed 0.71 among any age group at any time of night, with males aged 15–19 years the least protected at 0.54 (Figure 3C). A separate study in this village reported net usage at 91% in 2012 (J. Keven, L. Reimer, M. Katusele, G. Koimbu, R. Vinit, N. Vincent, E. Thomsen, D. Foran, P. Zimmerman, and E. Walker, submitted).

### Exposure

**Figure 4.**
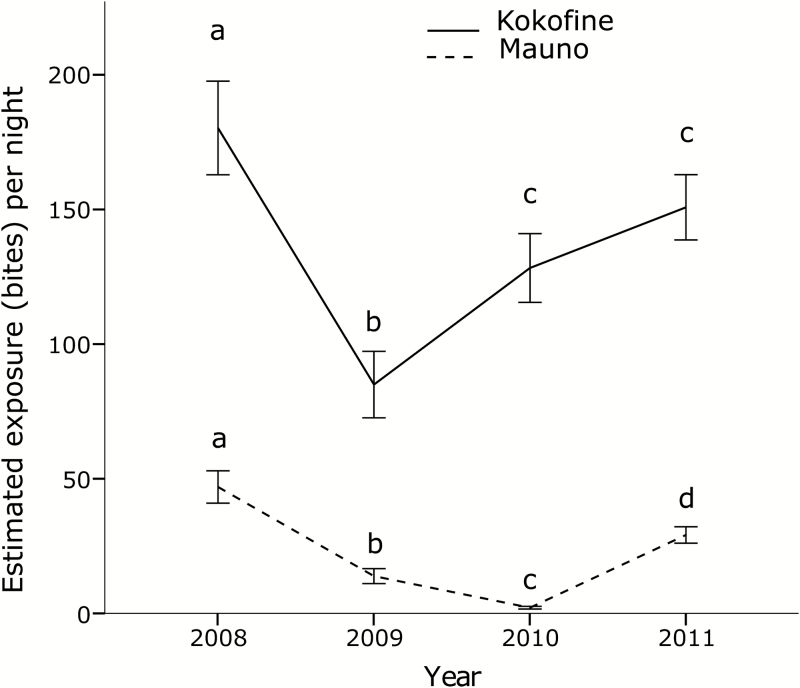
Total estimated exposure to bites for a non–net user before bednets (2008) and after bednets (2009–2011) in Kokofine and Mauno. Years sharing the same letters were not statistically different using a Kruskal–Wallis test with pairwise comparisons.

**Figure 5.**
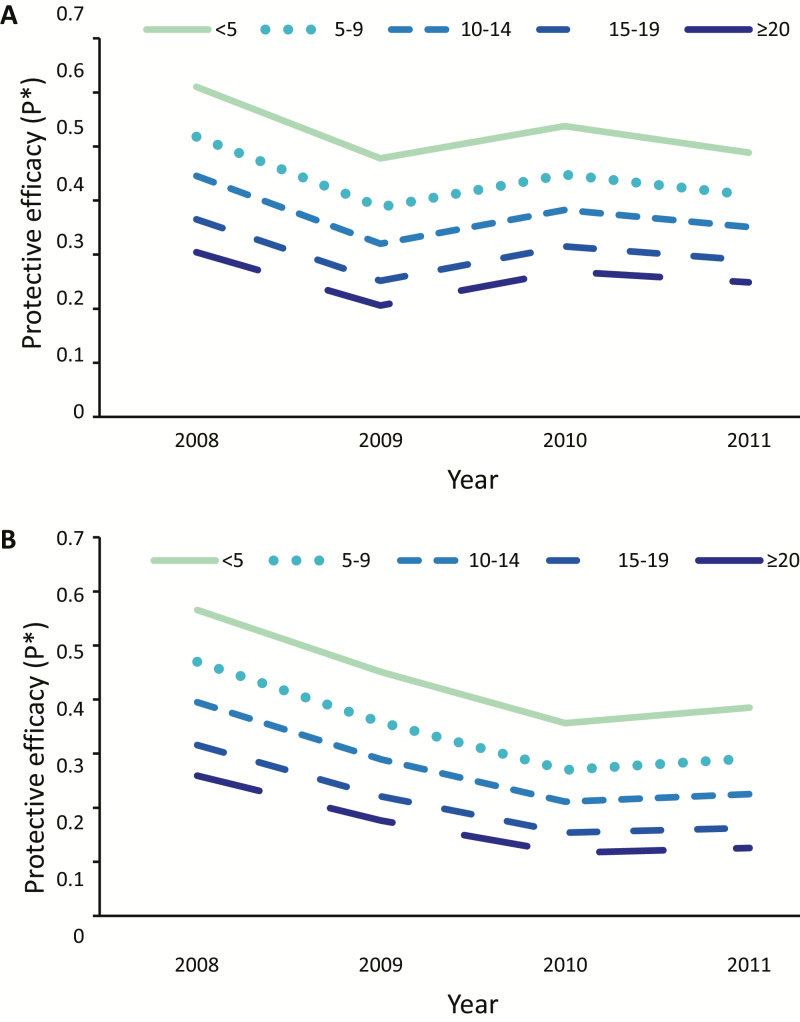
Protective efficacy (P*) by age group in Kokofine (*A*) and Mauno (*B*).

After a significant decrease in exposure between 2008 and 2009 in both villages (*P* < .001 for Kokofine and Mauno), there was subsequently a significant increase from 2009 to 2011 in Kokofine and from 2010 to 2011 in Mauno (Figure 4). Shifts to earlier bite exposure were observed in both villages (Supplementary Figure 3). Within each year, the estimated proportion of exposure occurring inside (π_i_) and the protective efficacy against bites (P*) was significantly greater in younger age groups (*P* < .001 for all years). Within each age group, there was a decrease in π_i_ and P* after LLINs were distributed, and the decrease was more pronounced in younger age groups (Figure 5; for π_i_: <5, *P* < .001; 5–9, *P* = .002; 10–14, *P* = .004; 15–19, *P* = .008; >20, *P* = .03; and for P*: <5, *P* = .001; 5–9, *P* = .003; 10–14, *P* = .006; 15–19, *P* = .01; >20, *P* = .02).

**Figure 6.**
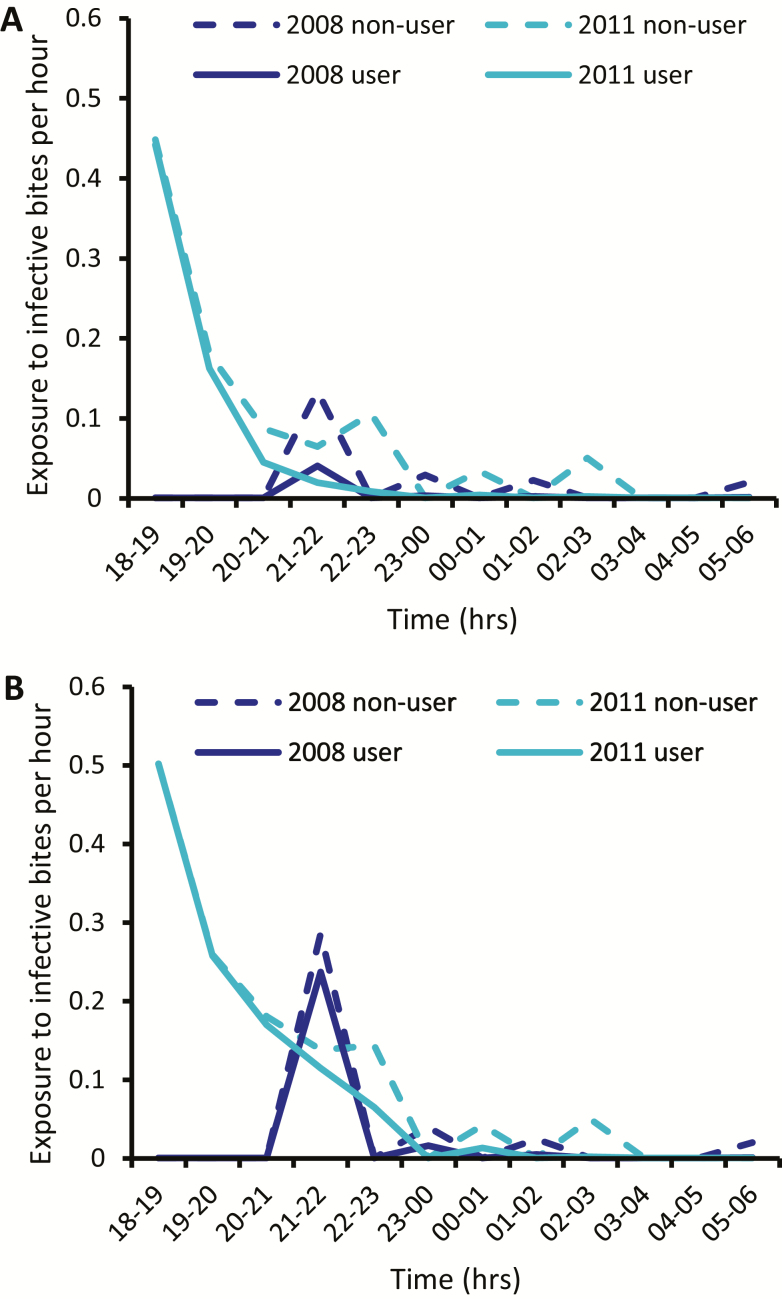
Estimated exposure to infective bites in children aged <5 years (*A*) and adults aged >20 years (*B*) in Kokofine. Exposure was estimated separately for bednet users and nonusers at the time of the distribution (2008) and 3 years later.

In Kokofine, the rebound in biting rates coupled with high sporozoite prevalence after LLIN distribution (in 2011) allowed us to quantify exposure to infective bites (Figure 6). In 2008, most exposure to infective bites occurred after 9 pm. In 2011, the majority of infective bites occurred during the first hour of collection, between 6 pm and 7 pm. In children aged <5 years, many infective bites would have been prevented by using a net in 2008; however, the protective efficacy of LLINs against infective bites (P*^f^) decreased drastically in 2011 because these bites were occurring before this age group went to bed. In adults aged >20 years, a similar but less pronounced decrease in P*^f^ was observed, primarily because this age group was always outside when infective mosquitoes were seeking a host. In 2008, P*^f^ was 0.78 in those aged <5 years and 0.30 in those aged >20 years. In 2011, P*^f^ had decreased to 0.30 and 0.15 in both groups, respectively.

### Human Infection Prevalence

**Figure 7.**
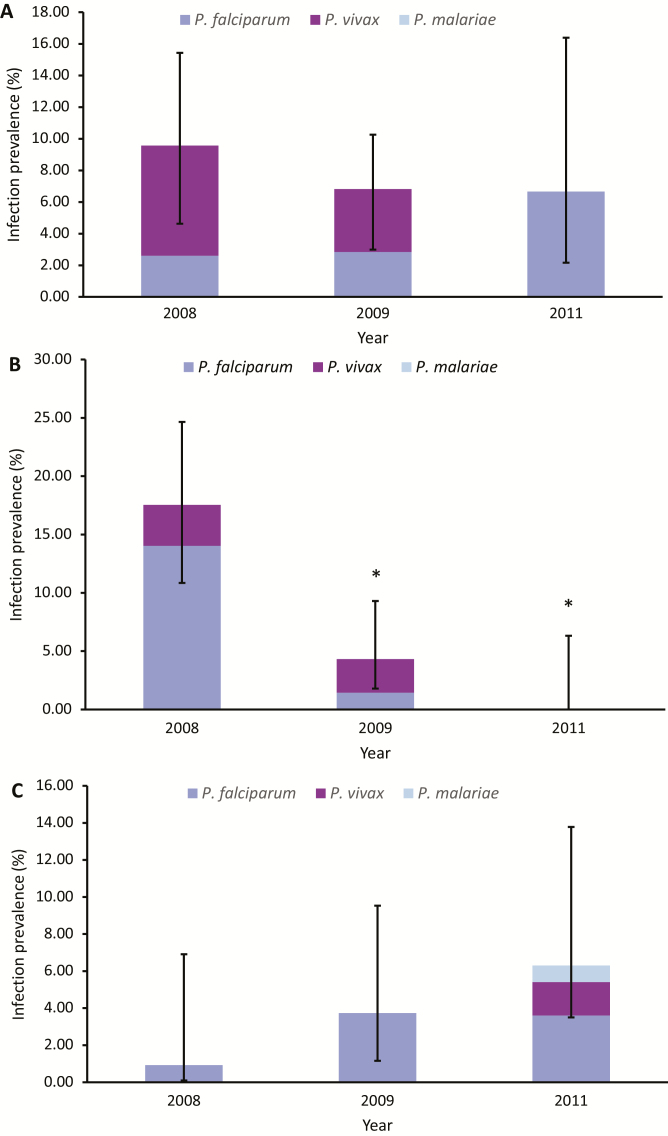
Human infection prevalence as detected by microscopy in Kokofine (*A*), Mauno (*B*), and Kesowai (*C*) villages before (2008) and after (2009 and 2011) a nationwide LLIN distribution in PNG. * indicates a significant change from 2008 (*P* < .05) using a chi-square test.

Data from the 2008 and 2009 malaria prevalence surveys have been published elsewhere [19] and are presented in greater detail here for context. Only Mauno showed a consistent and significant decrease in malaria prevalence across the 3 surveys. In Kokofine, there was no significant change in overall infection prevalence, and in Kesowai there was a nearly significant increase in prevalence from 2008 to 2011 (*P* = .058) (Figure 7).

## DISCUSSION

Our data show a clear and dramatic reduction in mosquito abundance in the first year after the LLIN distribution in both villages. However, a resurgence in mosquito abundance and exposure was documented between 2 and 3 years after intervention, coupled with a shift to significantly earlier biting. Based on the interaction between mosquito and human behavior, the protective efficacy of LLINs decreased during this resurgence, as more exposure to malaria vectors occurred before individuals were protected with a net. Besides behavioral resilience [24], 2 other factors may have contributed to the documented resurgence in mosquito abundance. First, physiological resistance to insecticides has also been shown to reduce intervention efficacy [25]. However, resistance is absent in members of the *An. punctulatus* group (the species group to which *An. farauti* 4 belongs) in PNG [26], and susceptibility has been confirmed from the Sausi region post-LLIN distribution (M. Katusele, unpublished data). This demonstrates that behavioral resilience may compromise intervention efficacy in a mosquito population that is fully susceptible to insecticide. Second, changes in bednet usage over time could limit the community effect of nets; however, usage increased over the study period. Furthermore, used nets from local communities retain the insecticidal effect against *An. farauti* for 5 years [27].

This is the first study to quantify human malaria infection prevalence in the context of shifting mosquito behaviors after an LLIN distribution. Malaria prevalence in humans decreased in only 1 of 3 villages, the village with the lowest biting rates, demonstrating the limited efficacy of nets to prevent disease transmission. Although the shifts in biting times would contribute to the limited epidemiological impact of the intervention, there are other factors that may have also played a role. Artemisinin combination therapy was only rolled out to the Sausi health center in the last quarter of 2011, which means that the population may have been receiving inadequate treatment. Treatment failures with the previous combination of chloroquine and sulphadoxine-pyrimethamine reached 18.5% in children with *P. falciparum* in PNG [28]. Migration of individuals into the study communities from areas of higher malaria burden may have also been a factor. Regardless, shifts in biting times have been documented in other mosquito populations in PNG [9]. As such, it will be important to continually monitor the epidemiological impact of LLINs in other areas where changes in mosquito behavior have been observed.

This is also the first study to quantify age-stratified exposure to bites of malaria vectors by taking into account the behaviors and sleeping patterns of each age group. Both the proportion of indoor bite exposure (π_i_) and the protective efficacy of LLINs against bites (P*) is greater in younger age groups, due to earlier sleeping patterns. This results in protection by an LLIN for a greater proportion of the entire period of exposure, which is a positive finding because this group is the most at risk for severe disease [29]. In contrast, the protective efficacy in adults is quite low (approximately 0.35 in both villages at the time of distribution) due to their greater outdoor activity patterns in the early hours of the night. Adults will continue to act as a reservoir of gametocytes, and LLINs may therefore have little impact on transmission reduction at the community level. This level of personal protection against bites is similar to that seen in other areas of the South Pacific [16].

The true protective efficacy of nets (against bites [P*] and infective bites [P*^f^]) decreased in both villages after LLINs were distributed. The reduced efficacy due to shifts in host-seeking times is a phenomenon that has been observed in other studies. In *An. funestus*, a shift to early morning feeding in southern Benin did not result in compromised efficacy because P* remained >80% [13]. In *An. farauti s.s.,* a shift to early evening feeding in the Solomon Islands did reduce P* [16]. However, individual mosquitoes showed no fidelity to biting time or location, and malaria burden continued to decline [14]. The authors hypothesized that over the course of several gonotrophic cycles, the likelihood of exposure to an LLIN before the end of the *Plasmodium* extrinsic incubation period still remained high [15]. Unlike the studies described above, our study suggests that the shift to early evening feeding in *An. farauti* 4 is epidemiologically significant—the estimate of the annual entomological inoculation rate in Kokofine was 827 infective bites per person per year in 2011, more than double the estimate of 343 in 2008. In addition, our analysis indicated that individuals were less protected from infective bites in 2011 than they would have been in 2008 due to the time infective mosquitoes were collected.

The underlying mechanism for the shift in biting times observed in this vector population is currently unknown. Biting behavior in anophelines appears to be a heritable trait because shifts in host-seeking behavior in the Solomon Islands during the dichlorodiphenyltrichloroethane spray campaign of the 1970s [3] remain to this day [16]. However, additional evidence for population-level selection for behavioral resistance is lacking [7]. Today, populations of *An. farauti s.s.* in the Solomon Islands are homogenous in their host-seeking behavior: subpopulations exhibiting different feeding preferences do not exist [14]. In addition, the genes responsible for the variation in feeding behaviors in malaria vectors have yet to be identified [30]. Additional hypotheses for the mechanism include associative learning [24] and delayed host-seeking due to unsuccessful attempts the previous night [31].

The sampling scheme used in this study had several limitations. First, human landing catches were not performed before 6 pm. This may have resulted in significant undersampling of the biting population after LLIN distribution. Second, the ratio of indoor to outdoor biting rates was measured during 1 collection period after LLIN distribution. The high degree of exophagy measured here is consistent with reports of *An. farauti* 4 in neighboring Papua, Indonesia [18], as well as other members of the *An. farauti* complex in the Solomon Islands [32]. Decreases in endophagy have been observed following indoor interventions [3, 4], which we are unable to capture in our study design. If early biting mosquitoes were undersampled or if the population experienced a shift in endophagy, the analysis would have underestimated the decreases in personal protection. Third, collections were performed in 2 weeks in 2008, 2009, and 2010 and 6 weeks in 2011, which may have highlighted week-to-week variation and obscured long-term trends.

Indoor interventions such as LLINs have contributed greatly to the reduction in malaria transmission over the last 15 years [1] and continue to provide significant community protection even in cases where shifts in biting behavior have been observed [6, 11, 12, 14, 15]. Our study highlights that in an area of high vector density and intense year-round transmission, shifts in biting behavior can have detrimental impacts on the personal protection provided by LLINs as well as community-wide transmission. Shifts to earlier biting after the bednet distribution resulted in greater exposure to infective bites, in net users and nonusers alike. The intervention achieved a reduction in malaria prevalence in only 1 of 3 villages studied despite high usage rates and net efficacy. Additional tools targeting outdoor and early biting mosquitoes will be necessary to control malaria and prevent a resurgence of transmission.

## Supplementary Data

Supplementary materials are available at *The Journal of Infectious Diseases* online. Consisting of data provided by the authors to benefit the reader, the posted materials are not copyedited and are the sole responsibility of the authors, so questions or comments should be addressed to the corresponding author.

## Supplementary Material

FigureS1

FigureS2

FigureS3

## Notes

***Acknowledgements.*** We acknowledge the Papua New Guinea Institute of Medical Research MalCon survey teams for the net use surveys and blood sample collection and Muker Sakur and Lemen Kilepak for assisting with mosquito collections. We would like to thank the communities in the Sausi region of Madang Province for participating in this study. We would also like to thank Dr Geraldine Foster and Professor Hilary Ranson for helpful comments on an early draft of the manuscript.

***Financial support*.** This work was supported by the Global Fund to Fight AIDS, Tuberculosis and Malaria (grant number PNG-304-G01-M).

***Potential conflict of interests*.** All authors: No reported conflicts. All authors have submitted the ICMJE Form for Disclosure of Potential Conflicts of Interest. Conflicts that the editors consider relevant to the content of the manuscript have been disclosed.
