# Supplementary material for: Mosquito Behavior Change After Distribution of Bednets Results in Decreased Protection Against Malaria Exposure
Source: J Infect Dis. 2016 Dec 22;215(5):790–7. doi: 10.1093/infdis/jiw615 (PMC5388271; doi:10.1093/infdis/jiw615)
Supplement: FigureS1 [file jiw615_suppl_FigureS1.pdf]

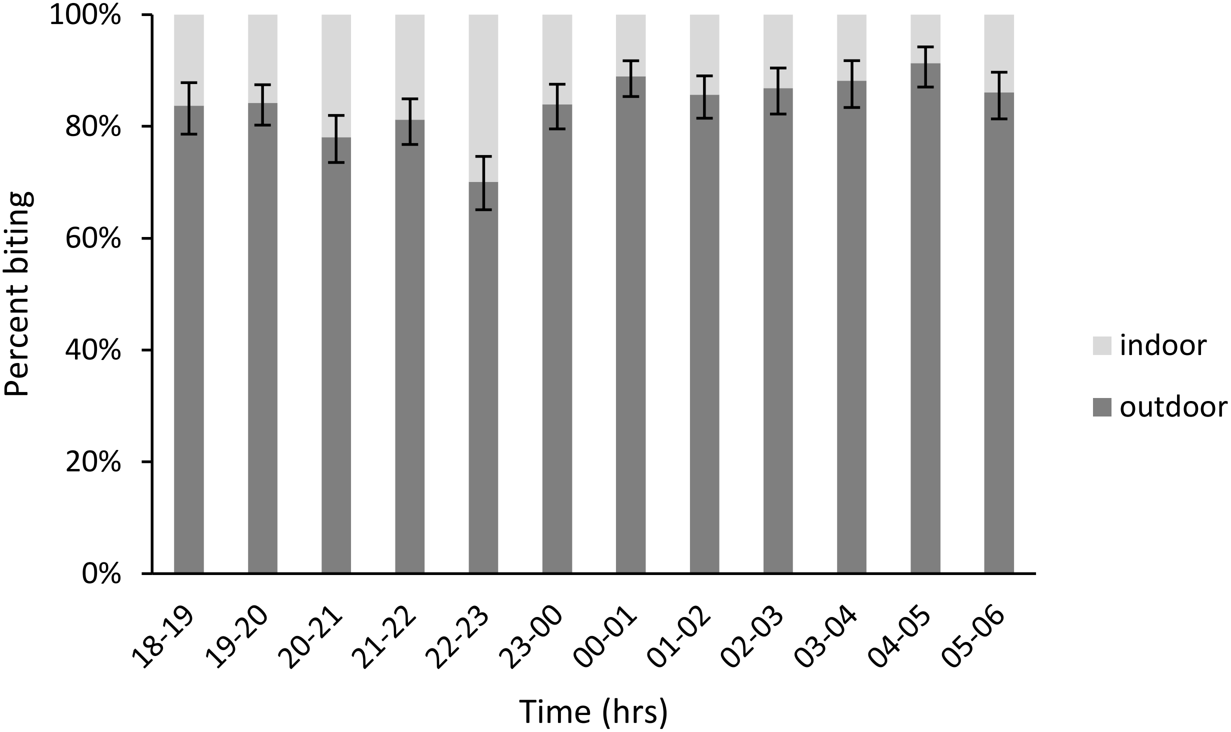

Figure S1. Percent of bites occurring inside and outside as measured by paired indoor and outdoor human landing catches. Error bars designate 95% confidence intervals
