# Supplementary material for: Mosquito Behavior Change After Distribution of Bednets Results in Decreased Protection Against Malaria Exposure
Source: J Infect Dis. 2016 Dec 22;215(5):790–7. doi: 10.1093/infdis/jiw615 (PMC5388271; doi:10.1093/infdis/jiw615)
Supplement: FigureS2 [file jiw615_suppl_figureS2.pdf]

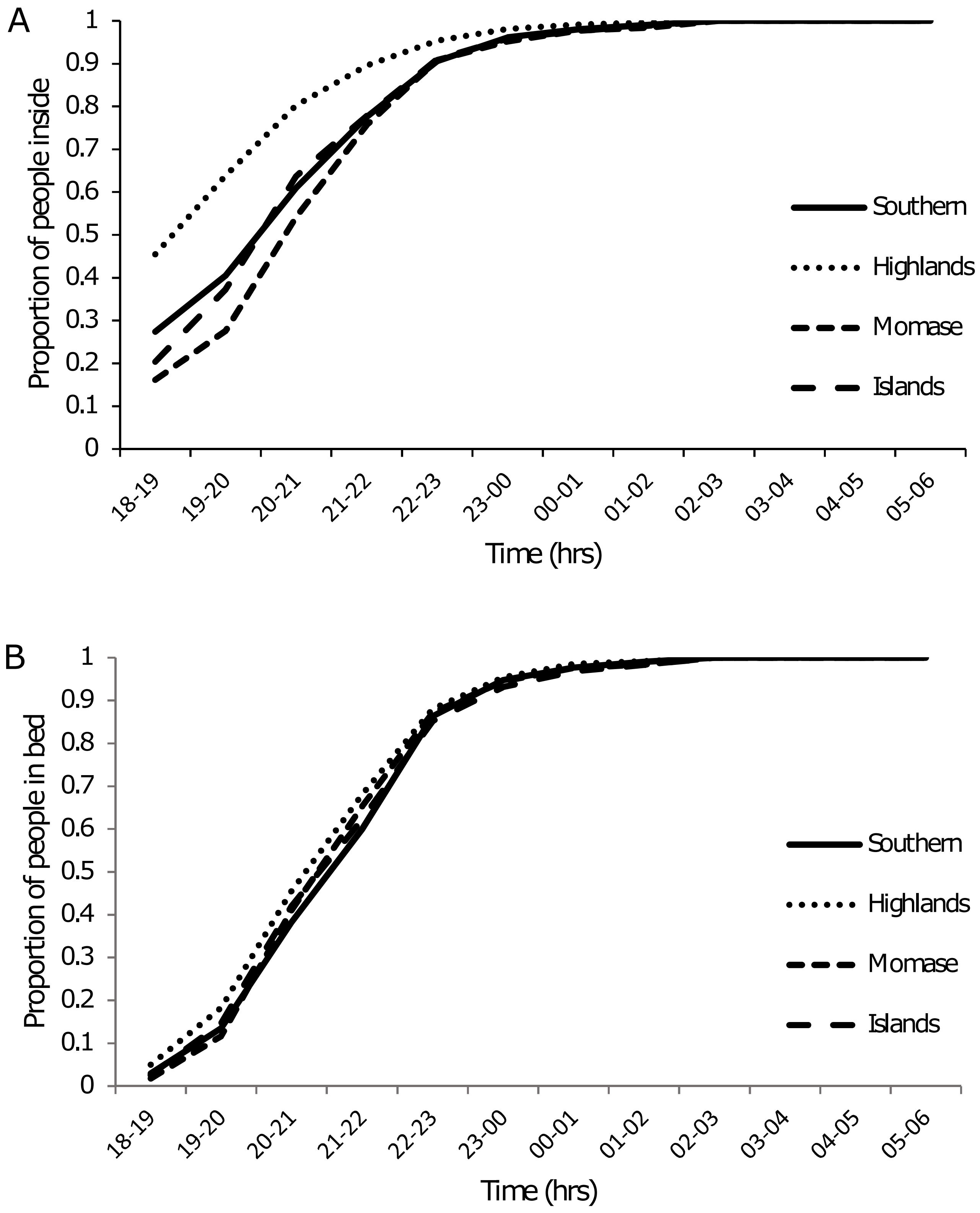

Figure S2. Proportion of individuals inside (A) and in bed (B) from 1800-0600 hrs in the 4 regions of Papua New Guinea.
