# Supplementary material for: Mosquito Behavior Change After Distribution of Bednets Results in Decreased Protection Against Malaria Exposure
Source: J Infect Dis. 2016 Dec 22;215(5):790–7. doi: 10.1093/infdis/jiw615 (PMC5388271; doi:10.1093/infdis/jiw615)
Supplement: FigureS3 [file jiw615_suppl_FigureS3.pdf]

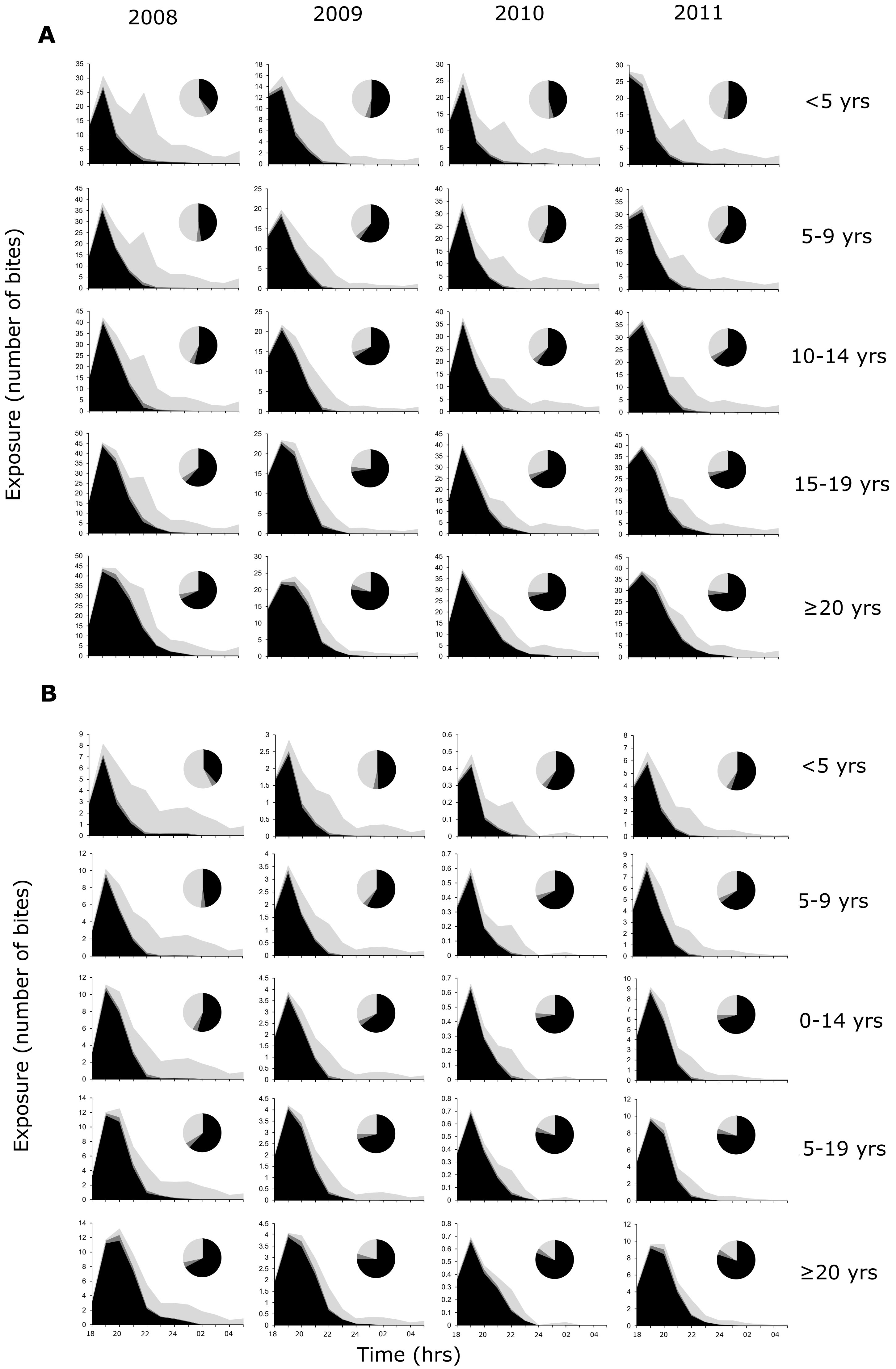

Figure S3. Exposure to bites occurring outdoors (black), indoors despite using an LLIN (dark grey), and indoors that is prevented by using an LLIN (light grey) in Kokofine (A) and Mauno (B) villages. Inset pie graphs show the same data as the stacked line graphs, but in proportions (the sum of both grey slices = πi)
